# How Many Mammalian Reovirus Proteins are involved in the Control of the Interferon Response?

**DOI:** 10.3390/pathogens8020083

**Published:** 2019-06-21

**Authors:** Delphine Lanoie, Simon Boudreault, Martin Bisaillon, Guy Lemay

**Affiliations:** 1 Département de microbiologie, infectiologie et immunologie, Université de Montréal, Montréal, QC H3C 3J7, Canada; delphine.lanoie@umontreal.ca; 2 Département de biochimie, Université de Sherbrooke, Sherbrooke, QC J1E 4K8, Canada; simon.boudreault@usherbrooke.ca (S.B.); martin.bisaillon@usherbrooke.ca (M.B.)

**Keywords:** virus, reovirus, interferon, viral proteins

## Abstract

As with most viruses, mammalian reovirus can be recognized and attacked by the host-cell interferon response network. Similarly, many viruses have developed resistance mechanisms to counteract the host-cell response at different points of this response. Reflecting the complexity of the interferon signaling pathways as well as the resulting antiviral response, viruses can—and often have—evolved many determinants to interfere with this innate immune response and allow viral replication. In the last few years, it has been evidenced that mammalian reovirus encodes many different determinants that are involved in regulating the induction of the interferon response or in interfering with the action of interferon-stimulated gene products. In this brief review, we present our current understanding of the different reovirus proteins known to be involved, introduce their postulated modes of action, and raise current questions that may lead to further investigations.

## 1. Introduction

Mammalian Orthoreoviruses, hereafter referred to as “reovirus”, are members of the Reoviridae family of viruses harboring a segmented double-stranded RNA genome. The reovirus has attracted a lot of attention in the last few years, since it is currently under study as a possible oncolytic agent. The interferon response appears quite relevant in this context, since cancer cells are often devoid of this pathway or altered in the response induced [1,2,3].

In this review, we mainly focus on what is currently known about the viral determinants that reovirus has developed to counteract the interferon response at different levels. We first briefly examine the reovirus multiplication cycle leading to the synthesis of viral nucleic acids that could be detected by the cells and how this could lead to induction of the antiviral response and the probable interferon-induced antiviral products involved. Then, each reovirus protein that is currently known as affecting either this induction or the sensitivity of the virus to the resulting antiviral response is examined in detail. Part of this manuscript was presented in French by D.L. as partial fulfillment for the MSc degree in Microbiology and Immunology at Université de Montréal.

## 2. Brief Overview of Reovirus Multiplication Cycle

Herein, we briefly present the aspects that are most pertinent in the context of the interferon response to reovirus. The readers are invited to consult an extensive book chapter on the whole viral multiplication cycle in [4] for further details.

### 2.1. Virus Entry in the Host Cell

Reovirus is a non-enveloped virus with a capsid made of two concentric layers of proteins. The viral particles can bind to the host cell, generally by means of its trimeric spike protein σ1. This cell-binding viral protein interacts with a first glycan receptor—sialic acid in most commonly used serotype 3 strains, such as the Dearing strain. Binding to a protein receptor of higher affinity, such as junctional adhesion molecule (JAM-A), on epithelial cells generally follows, although other receptors can be found on other cell types. Consecutive to host-cell binding, the viral particle is internalized, and the outer capsid is gradually digested, generating intermediates known as infectious subviral particles (ISVPs) and allowing the viral particle to cross the endosomal membrane. Infectious subviral particles can also infect cells by an entry route bypassing the requirement for endocytosis. Ultimately, either entry route is followed by the release of the inner capsid, known as the core structure, in the cytoplasm. It is this last form of the viral particle that is transcriptionally active and results in the synthesis of viral mRNA to pursue the multiplication cycle. For a more thorough review on early events related to viral entry, the reader is referred to some excellent reviews [5,6,7,8].

### 2.2. Transcription, Translation, and Replication of the Viral Genome

The core in the cytoplasm of the infected cell is responsible for viral mRNA synthesis and capping. The newly synthesized mRNA is extruded through turrets at the surface of the viral core and translated by the host-cell machinery to generate all viral proteins, eight structural proteins and four nonstructural ones. Each of the ten genomic segment encodes one protein except for the M3 (μNS and μNSC from two in-frame initiation codons) and S1 (σ1 and σ1s from two initiation codons in two different reading frames). Viral factories are produced in the infected cells where assembly of the progeny viral cores proceeds. Although details on the mechanisms of nucleic acids packaging are still somehow controversial, the current model indicates that one copy of each of the ten mRNA is packaged before synthesis of the complementary strand to generate the viral double-stranded RNA (dsRNA) genome. The outer capsid is then added, coinciding with the arrest of transcriptional activity. For a detailed review on transcription and translation during the viral multiplication cycle, the reader is referred to a recent review [9].

## 3. Reovirus and the Interferon Signaling Network

**Figure 1:**
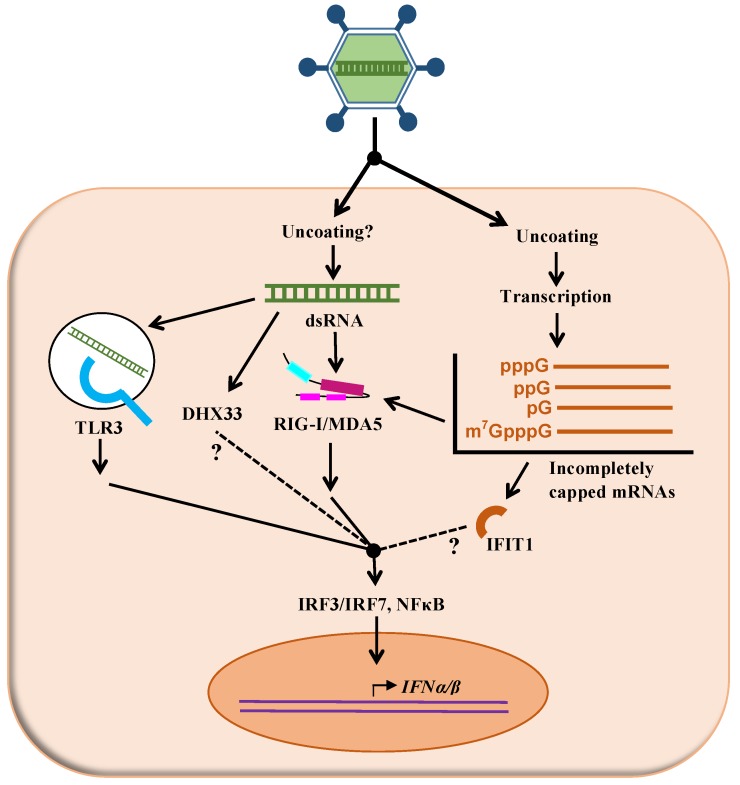
Putative mechanisms of recognition of reovirus by the interferon signaling network. As described in the text, different cellular proteins (TLR3, DHX33, RIGI, MDA5, IFIT1) are suspected to recognize either the viral double-stranded RNA genome (dsRNA) or different forms of incompletely capped mRNA generated by transcription of the viral genome. Following this recognition, adaptor molecules and signaling events, some of them only partly understood, result in increased transcription of interferon and ultimately interferon-stimulated genes (not shown on this figure).

Considering its mode of entry in the cell, it is postulated that reovirus genetic material could be recognized by various sensors of the innate immune response in order to activate the interferon response. In the present review, we focus on the role of viral proteins in the control of the interferon response and the ultimate impact on interferon sensitivity of the virus. However, it seems appropriate to briefly describe first how the cells recognize reovirus infection to initiate the response; this is also summarized in Figure 1.

A virus possessing a dsRNA genome, such as reovirus, could theoretically be recognized inside the endosomes by Toll-like-receptors-3 (TLR3) [10]. In the cytoplasm, viral RNA could also be detected by RIG-I-like receptors (RLRs). In fact, it was shown that the reovirus genome by itself can be recognized by both RIG-I and MDA5 [11]. These were described as being able to recognize double-stranded RNA of different lengths, or harboring different 5’ ends [12,13]. It is not clear if such dsRNA molecules could actually become available for recognition by the sensors during the viral replication cycle; although possible leakage of the viral genome from the viral particle has been proposed in the past [14], it does not seem that there is clear experimental evidence to support this idea. Diphosphorylated 5’-ends present on the minus strand of the viral dsRNA genome are also a potent pathogen-associated molecular pattern (PAMP) recognized by RIG-I [13]; however, again, it is not clear if these ends are actually exposed in the infected cells under normal infection conditions.

Another potentially important aspect for sensor recognition is the presence or the absence of a cap structure that could protect the viral mRNA against recognition as non-self RNA. In particular, the 2’-O methylation of the first nucleotide on the mRNA chain is clearly of importance as an evasion mechanism by viruses to escape the antiviral innate immune response. In fact, when various viruses lose this function, they become more sensitive to detection by the innate immune response or to the antiviral effect of this response. This is exerted through the action of interferon-stimulated factors, mainly due to the action of IFIT proteins [15,16,17,18,19]. Interestingly, it has been reported that part of viral mRNA is devoid of a cap structure late in infection, rather harboring a monophosphate end, although the transient presence of a diphosphorylated end is possible [20,21,22] (for a recent review on reovirus mRNA structure and synthesis, see [9]). Finally, in the related rotavirus, it was more recently shown that a fraction of the RNA is not completely capped, and this seems to be responsible for recognition by the sensor RIG-I [23].

Interestingly, different experimental evidence indicates that virions and ISVPs differ in their ability to induce the interferon response [24,25,26]. Virions do appear as stronger inducers, and differences between viral strains could be at least partly abolished when cells are infected by ISVPs rather than virions, suggesting that early uncoating events are somehow involved in determining the extent of interferon induction. Direct transfection of viral cores was also shown to abolish differences in interferon induction between different serotypes; some of these data were interpreted as an indication that differences in viral genome “delivery” are involved in induction of interferon [26], although the exact underlying mechanism remains elusive. 

Expression of interferon-stimulated genes (ISGs) is strongly reduced in cells that lack both RIG-I and MDA5 [11]. RIG-I seems especially important in the context of infection [11,13,27,28,29]. Nevertheless, although these studies suggest that the role of TLR3 is likely marginal compared to that of RIG-I, it might not be the case in all cell types [30,31]. Moreover, differences in the specific properties of viral strains used in these studies could explain some apparently divergent results. Finally, the helicase DHX33 was suggested as a possible additional sensor in myeloid dendritic cells [32]. Clearly, additional work using cells and viruses harboring specific changes in the same genetic background are needed to fully comprehend the likely multiple viral and cellular determinants underlying reovirus sensing by the host cells.

## 4. Cellular Antiviral Interferon-Stimulated Genes Involved in Reovirus Resistance

The dsRNA-dependent protein kinase (PKR), also known as eIF2AK2 (or alpha, α) kinase, is the most clearly established product of an interferon-stimulated gene to be involved in reovirus resistance upon interferon treatment [33]. The antiviral effect of PKR on reovirus has also been the topic of extensive reviews in the past [34,35,36]. Once its expression is induced by interferon, PKR is activated by RNA (either double-stranded or single-stranded with extensive secondary structure) and can inhibit protein synthesis due to phosphorylation of the alpha subunit of translation initiation factor 2 (for a review of PKR antiviral function, see [37,38]). PKR is also likely involved in the inhibition of host-cell protein synthesis during viral infection, a phenomenon that is not necessarily directly due to the interferon response [39]. Also, other evidence indicates that a certain level of PKR activity could be beneficial to reovirus replication [40]. The complex role of PKR in reovirus replication has also been recently reviewed [9]. Finally, the true importance of PKR and the required level to achieve a balance between its negative and its positive effects on viral replication may well vary among cell types [41].

There have been few reports indicating that other known ISGs can exert an effect on reovirus replication at different stages of the viral replication cycle. In fact, it has been shown that the 2’-5’A activator of RNase L is found in some reovirus-infected cells [42]. Cleaved RNA, most likely due to the activation of RNase L, is also found in these cells. Furthermore, recent data indicate that RNase L has an important role in regulating PKR activity [43]. The myxovirus resistance protein 1 (known as Mx1 or MxA), a well-known ISG, was also shown to be able to strongly affect reovirus replication [44]. However, its role in the context of the interferon response per se was not directly examined, for example, by looking at its effect on viral replication following interferon treatment. Also, although their effect appears somewhat limited, both the interferon-inducible transmembrane protein IFTM3 and the enzyme cholesterol-25-hydroxylase were reported to affect viral entry following endocytosis at the level of the endosomal compartment. As a result, only virions are affected, while ISVPs are resistant [45,46].

Altogether, it thus appears that multiple interferon-induced antiviral proteins could exert an effect against reovirus. Interestingly, these different ISGs were shown to be up-regulated at both the mRNA and the protein levels in infected cells [47,48]. Some of these are likely to be somewhat specific to some cell types, and a combination of more than one of these antiviral proteins is probably involved in the final effect of interferon in any given cell types.

## 5. Viral Inhibition of the Antiviral Interferon Network

In response to the cellular defense mechanisms, many viruses encode proteins whose function is to protect them against the cellular antiviral response, especially the response related to the interferon signaling network. A complete description is beyond the scope of the present review, and the reader is referred to many recent and excellent reviews of the topic [49,50,51,52,53,54].

Briefly, one can envision three different overall strategies that a virus could use to interfere with the pathways of the interferon antiviral network. Firstly, the virus could protect itself against recognition by the host cell’s sensors, thus preventing induction of the response. Secondly, the virus could interfere with adaptor molecules involved in the interferon response network, either directly or by inhibiting their signaling. Finally, the virus could interfere with either recognition by the interferon-stimulated antiviral gene products (ISGs) or interfere with the action of one or more of these gene products. 

## 6. Reovirus Proteins Involved in the Control of the Interferon Response

Considering the previous observations of the many sensors and ISGs potentially involved in the antiviral effect of interferon on reovirus, it was somehow expected that more than one viral protein could be involved in the control of the interferon response. While, for some of these proteins, the mode of action is relatively well-known, for others, additional work is necessary to better comprehend their mode of action. These different reovirus proteins are each briefly reviewed for their probable role in either the control of induction of the interferon response or the sensitivity of the virus to this response. For the sake of simplicity, the location and the function of reovirus proteins are summarized in Table 1. Their functions, if any, in the control of the interferon response are summarized in Table 2.

### 6.1. The σ3 Protein

The σ3 protein (encoded by the S4 gene), a major component of the outer viral capsid, has long been proposed to exert an important role in reovirus’s control of the interferon response, more specifically at the level of sensitivity to the interferon-induced dsRNA-dependent protein kinases (PKR) (previously reviewed in [9,34,35,55]). The σ3 protein possesses a long-known ability to bind double-stranded RNA. In addition to being an inducer of the interferon response, as discussed in Section 3, double-stranded RNA also allows dimerization of PKR, resulting in activation of its activity (for a review of PKR activity and function, see, among others, [37,38]). This sequestration of dsRNA by σ3 should thus interfere with recognition of the viral genetic material by PKR or other cellular determinants that possess an affinity for dsRNA, such as RIG-I and MDA. This interference with PKR action thus allows the cell to pursue synthesis of the viral proteins in the infected cell.

An 85 amino acids long domain in the carboxyl-terminal portion of σ3 encompassing two basic amino acids motifs was initially identified in biochemical assays as being responsible for the binding to viral RNA [56,57,58,59] (also reviewed in [9,34,35,55]). Later on, the structure of σ3 was determined by X-ray crystallography and revealed that the protein can form a dimer by itself [60]. The σ3 protein also interacts with μ1 to form the heterohexamer that makes the bulk part of the outer capsid [61]. More recent data confirmed the existence of the σ3 dimer in infected cells as well as the transition from dimer to heterohexamer due to the action of the cellular chaperone TriC [62]. This suggests a transition from the regulatory role of σ3 through its binding to dsRNA to the structural role exerted by the σ3-μ1 heterohexamer. It should also be stressed that a great deal of experimental evidence has already shown that the binding of σ3 to RNA or to μ1 is mutually exclusive [24,63,64]. A second model for the binding of σ3 to dsRNA was thus proposed, wherein the basic surface formed by the dimer form of σ3 is responsible for the binding rather than binding to the two basic domains of a single σ3 monomer [55,60]. Interestingly, some critical residues, such as lysine 293, are included in both the basic amino acids motifs and the basic surface of the homodimer and are thus common to both models.

During reovirus infection, stable levels of σ3 are eventually reached, while a gradual decline in the proportion of σ3 that is bound to dsRNA is observed at later times in infection [65]. This further support the idea that the role of σ3 early in infection would be to sequester dsRNA, thus limiting PKR activation. Dissociation from dsRNA and association with μ1 occurs later, depending on chaperone activity, in order to form the outer capsid of newly assembled virions. Interestingly, it has also been observed that the extent of co-localization between σ3 and μ1 in infected cells varies between viral isolates and correlates with increased inhibition of host-cell protein synthesis [66]. This was interpreted as an increased activity of PKR when σ3 is bound to μ1, thus resulting in protein synthesis inhibition, again consistent with the model, even though the role of PKR in inhibition of host-cell protein synthesis remains debated (for a recent review of this aspect, see [9]).

Different studies have shown the ability of σ3 to replace the protein involved as the interferon-controlling determinant in different viruses and thus complement these viruses that are otherwise defective [67,68,69,70]. However, in most cases, these experiments examined the ability of σ3 to complement virus replication, an effect that was attributed to inhibition of the interferon response. Interferon induction or viral resistance to interferon treatment was not directly examined in these studies, raising doubts to their true significance in the context of interferon response as such. At this point, the importance of σ3 in the control of interferon response in reovirus-infected cells thus remains to be directly demonstrated. The introduction of plasmid-based reverse genetics [71] (reviewed in [72,73]) could first allow to clarify the nature of the amino acid motifs involved in the binding of σ3 to dsRNA binding in reovirus-infected cells. Thereafter, the importance of this binding in the control of interferon induction and/or sensitivity to interferon could be more clearly examined using viruses harboring relevant amino acids substitutions of σ3 in the same genetic background. 

### 6.2. The μ2 Protein

The μ2 protein (encoded by the M1 gene and found in the inner viral core) was first shown to be associated with induction of interferon type I α/β in cardiac myocytes as well as a concomitant sensitivity of the virus to this antiviral response [74]. Subsequent studies have shown that the amino acid at position 208 on μ2 is critical to repressing interferon signaling pathways. If proline is found at this position, as is the case on serotype 1 Lang (T1L) virus strain, induction of the interferon pathway is repressed; on the contrary, a serine at this same position, as in serotype 3 Dearing strains (T3D), abolishes this effect [74,75]. The μ2 protein is, in fact, able to alter the activity of the IRF9 transcription factor (interferon response factor 9) by inducing its unusual accumulation in the cell nucleus [76]. Interestingly, recent work also showed the polymorphism of μ2 at amino acid 208 among T3D virus stocks of different laboratories is partly responsible for the difference in interferon response between these stocks [77]. The exact reason for the importance of this amino acid remains to be determined, considering that it alters multiple properties of the protein, such as association with microtubules, as described below.

In addition, although three reovirus proteins harbor an immunoreceptor tyrosine-based activation (ITAM) motif, only that of μ2 (YXXLX_9_YXXL) is actually functional and appears responsible for NF-kB activation, which could result in interferon induction in some cell types [78]. It remains to be established if this alternative pathway leading to activated interferon network is more or less important than the more classical pathway using IRF3 (Figure 1).

It is well known that the μ2 protein plays multiple roles during the viral multiplication cycle. For example, it is known to be involved in the morphology of viral inclusions due to its interaction with microtubules [79,80,81]. This property seems to modify the efficiency of viral particles assembly and affects the percentage of infectious virions produced by infected cells [82,83]. Furthermore, μ2 can bind RNA and has a 5’-RNA triphosphatase activity and thus probably plays a role in the synthesis of the cap structure at the 5’ end of viral mRNA [84,85] (recently reviewed in [9]). It could be interesting to verify if one or more of these different properties could be related to the control of the interferon response. More recently, it was observed that reovirus infection could also affect the cellular mRNA alternative splicing landscape [47]. It was proposed that the μ2 protein could be involved, since it interacts with the SRSF2 splicing factor [86]. Since it has been shown that some viruses do negatively regulate the interferon response through altered splicing events [87,88,89], this clearly deserves further study.

### 6.3. The μNS Protein

The non-structural protein μNS (encoded by the M3 gene) is both necessary and sufficient to form viral inclusions or factories, where the virus replicates its genome and assembles new virions [90,91,92] (recently reviewed in [93]). A recent study revealed that viral inclusions could help reovirus to avoid the innate immune response of its host cell [94]. In fact, in infected cells, the IRF3 transcription factor would be sequestered in cytoplasmic viral inclusions, thus interfering with induction of interferon. This trapping of IRF3 by μNS would be directly linked to the ability of the viral protein to form inclusions. When cells were infected with viruses harboring a μNS that was either truncated or harboring substitutions to prevent the formation of viral inclusions, a significantly higher proportion of IRF3 was found to be localized to the nucleus. This, in turn, promoted interferon production and activation of signaling pathways leading to ISG synthesis [94].

Interestingly, viral inclusions were also recently reported to trap the stress granule protein G3BP1 [95], while μNS was also found in stress granules during infection [96]. Stress granules are likely to have an impact on the innate immune response, including control of PKR, although our overall understanding remains limited (reviewed in [97,98]); most recent data suggest that G3BP1 can interact with RIG-I to promote the interferon response [99]. It is thus possible that μNS could indirectly affect the interferon response by promoting the formation of viral inclusions leading to trapping of G3BP1.

### 6.4. The λ2 Protein

The λ2 protein (encoded by the L2 gene) is responsible for the formation of the transcapsid turrets, cylindric structures each made of a homopentamer of the protein. These turrets allow anchoring of the homotrimeric σ1 protein forming the cell-binding spikes at the surface of the viral particle. In addition to this important structural role, the function of λ2 is largely enzymatic. This protein possesses a guanylyltransferase as well as both methyltransferase 1 and 2 enzymatic activities [100,101]. A single catalytic domain for guanylyltransferase activity and two putative methyltransferase domains were identified on the crystallographic structure of the protein [102] (reviewed in [9]). These enzymatic functions allow the synthesis of the cap structure at the 5’ end of viral messenger RNA. An initial study using classical gene reassortment studies showed that differences in λ2 could be linked to different sensitivity of the virus to IFN in murine cardiomyocytes [76]. More recently, the use of the reverse genetics approach allowed to demonstrate that the increased interferon sensitivity phenotype observed with a reovirus mutant was due to a single amino acid substitution in λ2 [103,104]. This substitution is found in the first methyltransferase domain of the protein (amino acid 434 to 691)—more precisely, at position 636. Due to this mutation being located in a methyltransferase domain, it was suggested that this enzymatic function is altered in the mutant. This could prevent adequate capping of viral mRNA, most probably the addition of the 2’-O-methyl group, an important determinant of interferon sensitivity, as mentioned in Section 3. 

Increased interferon sensitivity of the so-called T3D^K^ variant (Kobayashi, from the laboratory of Terence Dermody) of the wild-type virus compared to the T3D^S^ variant (Sandekian, from the laboratory of Guy Lemay) is also due in part to another difference in the λ2 protein; these two variants are due to differences in the wild-type viral stocks from the two laboratories. The amino acid substitution on λ2 is again found in the first methyltransferase domain at position 504 [77]. It is thus possible that these two substitutions can exert a similar effect. However, the substitution at position 636 exerts an effect by itself, while the 504 substitution requires an additional substitution in μ2 (the P208S change already described in Section 6.2). Since the exact mechanistic impacts of substitutions in λ2 remain to be established, it is also possible that these substitutions somehow affect the exit of the mRNA from the core structure through the pentameric λ2 turret.

Additional studies are needed to confirm or infirm these hypotheses, especially since the role of the first methyltransferase domain as either m^7^G or 2’-O methyltransferase remains somehow controversial [100] (reviewed in [9]). It should be possible to examine transcription and the 5’ end of the viral mRNA in order to determine if the amino acid substitution actually results in a change in synthesis of mRNA or methylation of the cap structure.

### 6.5. The λ1 Protein

Even though the role of λ1 in reovirus replication remains to be more firmly established, biochemical studies have shown that this protein harbors a nucleic acid binding motif and possesses both a helicase and 5’-RNA triphosphatase activity [85,105,106,107,108]. It is thus suggested that λ1 can be, among other functions, involved in replication of the viral genome, its transcription, or synthesis of the mRNA cap structure.

The strong interferon induction by the T3D^K^ variant of the wild-type virus compared to T3D^S^ is not only due to polymorphism at amino acid 208 of μ2, as described in Section 6.2, but also to the presence of a unique amino acid difference on λ1 (I500S). Additional work is needed to determine the mechanism behind this effect of λ1 polymorphism. Is λ1 acting to dampen the interferon response, as is believed to be the case for μ2, or is it somehow involved in inducing the interferon response to a different extent depending on virus strains?

Interestingly, variations in the levels of ATPase activity have been observed between strains of reovirus [109]. In fact, the T1L strain was shown to hydrolyze ATP more rapidly than T3D, and these variations could be potentially due to one or more of five amino acid differences in λ1 between the two strains [110]. Strikingly, while isoleucine is found at position 500 in the T3D strain used in this study, as in the T3D^K^ variant, a serine is rather found in T1L, as in T3D^S^. This suggests the possibility that the λ1 protein of T3D^S^ is more active than that of T3D^K^ and is similar to that of T1L; as a result, ATP levels are reduced upon infection by T3D^S^ compared to T3D^K^. A recently published study showed that ATP can exert a protective effect on cells in the context of viral infection [111]. Despite the fact that its exact mechanism of action in the context of viral infection remains unknown, studies suggest that the ATP receptor, P2X7, is essential to innate immune response [112]. The extracellular ATP released by infected cells could act as a danger signal and is active in vitro and in vivo against viruses such as vesicular stomatitis virus, Newcastle disease virus, and herpes simplex virus by increasing β-interferon production [111].

Altogether, these observations are consistent with the possibility that varying levels of interferon induction upon infection could be due to differences in ATPase activity of λ1 of the viruses. It could be interesting to determine if cells infected by T3D^K^ do actually release more extracellular ATP than cells infected by T3D^S^, as can be expected. Also, once again, since the λ1 protein was also proposed as a putative RNA triphosphatase responsible for the first step in cap synthesis (as for μ2), it is possible that cap synthesis is more efficient in T3D^S^ than in T3D^K^. It should be noted that the ATPase activity and the RNA triphosphate activity were assigned to the same catalytic site on the λ1 protein [107].

### 6.6. The σ1s protein

In the course of our work on the adaptation of reovirus to different host cells, the Vero cell-adapted virus (VeroAV) was obtained [113,114,115]. Although the virus does not induce more interferon, it turned out to be significantly more sensitive to interferon treatment. As mentioned above, a lower induction of interferon was observed in the presence of proline at position 208 on μ2 and/or in the presence of serine at position 500 on λ1. This was also observed for induction of various ISGs, such as ISG15, IFIT1, and MX1 [48]. Amino acids sequence of both μ2 and λ1 proteins of VeroAV that were unchanged at these critical amino acid positions compared to the parental virus was consistent with the low level of induction of interferon observed, as with the parental virus. 

Using plasmid-based reverse genetics, the interferon sensitivity phenotype of VeroAV was attributed solely to one of the two nucleotide substitutions found in its S1 gene. This substitution was responsible for both a Q78P amino acid change in σ1 and a N59H amino acid change in the small σ1s protein encoded in the second overlapping reading frame of S1 [115]. The second substitution observed in S1 resulted in a N198K amino acid change that was shown to be responsible for a better infectivity, especially on Vero cells, due to increased binding to sialic acids at the cell surface [114]. 

Interestingly, a former study showed that a mutant obtained following reovirus adaptation to murine erythroleukemia cells (MEL) also possessed two mutations in the S1 gene. In fact, this MEL-adapted virus did not produce the σ1s protein due to the presence of the stop codon at the start of the corresponding reading frame. The authors also noticed a second W202R amino acid change on σ1 [116]. Amino acids at this last position, as well as those of position 198 of VeroAV, are both part of the sialic acid binding domain [117,118]. It is thus tempting to speculate that introduction of a basic amino acid in either N198K or the W202R substitution could result an increased affinity of σ1 for sialic acid at the cell surface. This could somehow counterbalance the loss or the altered functional properties of σ1s. In fact, the σ1s-N59H substitution of VeroAV by itself or the introduction of a stop codon to abolish the expression of σ1s were both shown to decrease cell lysis of infected L929 cells [115]. It should also be mentioned that, during the establishment of viral persistence on Vero cells, the N198K substitution appeared before the Q78P/N59H substitution [113,114]. This could be explained if an increased infectivity due to the N198K substitution of σ1 is a prerequisite to allow subsequent changes altering the amount or the function of σ1s.

In accordance with the idea that σ1s could play a role in the ability of the virus to infect, a recent study showed that it is required for optimal synthesis of viral proteins, thus allowing better viral replication [119]. It was also observed that viral factories are altered upon infection by the knockout mutant, resulting in reduced assembly of infectious virions. The authors of this study indicate that σ1s is not involved in the control of induction of type 1 interferon response. Levels of β-interferon produced, as well as levels of STAT1, STAT2, and IFIT1 with the knockout virus mutant, were similar to those observed upon infection with a wild-type virus. However, they did not directly examine interferon sensitivity of the viruses. An absence of effect on induction of the interferon response upon deletion of σ1s is, however, consistent with our own data, despite increased interferon sensitivity with either the substitution mutant N59H or a complete knockout of σ1s [115].

The σ1s protein is responsible for G2/M cell cycle arrest observed in some viral strains [120,121]. Interestingly, recent data indicate a link between cell cycle arrest at G2/M and reduced interferon sensitivity of different viruses due to reduced induction of interferon-stimulated genes [122]. There is thus a possibility that σ1s interferes indirectly with the antiviral action of interferon by inducing the G2/M cell cycle arrest. However, a virus mutant defective in the ability to induce the cell cycle arrest was shown to be more resistant to interferon in vitro while being attenuated in vivo [121]. Clearly, once again, studies using isogenic viruses solely differing in σ1s should be undertaken. This should clarify if there is actually a functional link between cell cycle arrest and the interferon response in the context of reovirus infection both in vitro and in vivo.

## 7. Conclusions

In the last decade, progress in sequencing technology, site-directed mutagenesis, and the advent of plasmid-based reverse genetics has allowed the identification of molecular determinants of various phenotypic properties of reovirus. Classical genetics approaches to select viruses with distinct phenotypes combined with plasmid-based reverse genetics to identify and further study the molecular determinants involved have been instrumental in most of our recent progress. In this short review, emphasis was put on the recent identification of multiple reovirus determinants of induction and sensitivity to the antiviral interferon response. The apparent multiplicity of viral proteins involved is consistent with data obtained in the last few years with such diverse viruses as influenza, rotavirus, hepatitis C, and vesicular stomatitis virus, among others [123,124,125,126,127]. It appears that the control of the interferon response is somehow shaping the whole viral genome. In some cases, it has been shown that removal of the selective pressure conferred by the antiviral interferon network results in an overall reconfiguration of the viral genome, likely toward more effective viral replication or transmission.

Further work is clearly needed to understand the exact mode of action of the different reovirus proteins in different cell types, as well as *in vivo*, in order to better comprehend the real importance of these various viral determinants in viral replication and pathogenesis. A summary of the different reovirus proteins involved in either the control of induction of or the sensitivity to the antiviral interferon response as well as their putative mode of action is presented in Table 2. As described in this brief review, various mutants have been obtained that differ in either induction of or sensitivity to the interferon response. These will be very useful to pursue the study of interferon response. In addition, the powerful tool of reverse genetics could be used to further combine various viral determinants in these studies. In addition to a gain in fundamental understanding, these studies could likely contribute to better adaptation of the virus toward different cell types as a virotherapeutic oncolytic agent [128,129,130].

## Figures and Tables

**Table 1:** Biochemical and biological properties of reovirus proteins.

	Location in Virion	Biochemical Activity	Other Properties
λ1	Inner capsid	Helicase NTPase RNA triphosphatase	
λ2	Trans-capsid	Guanylyltransferase Methyltransferase (mRNA capping)	Presence in stress granules.
λ3	Inner capsid	RNA polymerase (transcription and replication)	
μ1	Outer capsid		Forms pores in endosomes. Forms heterohexamers with σ3. Role in cellular apoptosis.
μ2	Inner capsid	Helicase NTPase RNA triphosphatase	Binds to microtubules. Affects factory morphology. Partial nuclear distribution. Affects genome packaging?
μNS	Non-structural		Major component of factories. Scaffold for core assembly.
μNSC	Non-structural		
σ1	Outer capsid	Possible glycosidase activity	Host-cell binding moieties. Forms a homotrimer.
σ1s	Non-structural		Partial nuclear distribution. Role in cell cycle arrest. Increases viral proteins synthesis.
σ2	Inner capsid	dsRNA binding	
σ3	Outer capsid	dsRNA binding	Forms homodimer. Forms heterohexamers with μ1. Possible nuclear presence. Stimulates translation of late viral mRNA?
σNS	Non-structural	RNA binding Probable RNA chaperone	Role in formation of viral inclusions

**Table 2:** Reovirus proteins involved in the control of the interferon response.

	Role in Induction	Role in Sensitivity	Nuclear Presence	Postulated Mechanism
λ2	No	Yes	No	2’O-methylation of viral mRNA
λ1	Yes	No	No	ATPase activity? RNA capping?
μ2	Yes	Yes	Yes	nuclear trapping of IRF9
μNS	Yes	?	No	inclusion trapping of IRF3
σ1s	No	Yes	Yes	cell cycle arrest?
σ3	No?	Yes	Yes?	PKR inhibition by dsRNA binding

PKR: dsRNA-dependent protein kinases.
